# Superfluid Phase Transitions and Effects of Thermal Pairing Fluctuations in Asymmetric Nuclear Matter

**DOI:** 10.1038/s41598-019-54010-7

**Published:** 2019-12-06

**Authors:** Hiroyuki Tajima, Tetsuo Hatsuda, Pieter van Wyk, Yoji Ohashi

**Affiliations:** 1 Quantum Hadron Physics Laboratory, RIKEN Nishina Center, Wako, Saitama 351-0198 Japan; 2 Interdisciplinary Theoretical and Mathematical Sciences Program (iTHEMS), RIKEN, Wako, Saitama 351-0198 Japan; 3 Department of Physics, Keio University, Hiyoshi, Kohoku-ku, Yokohama 223-8522 Japan

**Keywords:** Astronomy and planetary science, Materials science, Physics

## Abstract

We investigate superfluid phase transitions of asymmetric nuclear matter at finite temperature (*T*) and density (*ρ*) with a low proton fraction (*Y*_p_ ≤ 0.2), which is relevant to the inner crust and outer core of neutron stars. A strong-coupling theory developed for two-component atomic Fermi gases is generalized to the four-component case, and is applied to the system of spin-1/2 neutrons and protons. The phase shifts of neutron-neutron (nn), proton-proton (pp) and neutron-proton (np) interactions up to *k* = 2 fm^−1^ are described by multi-rank separable potentials. We show that the critical temperature ${{\boldsymbol{T}}}_{{\bf{c}}}^{{\bf{n}}{\bf{n}}}$ of the neutron superfluidity at *Y*_p_ = 0 agrees well with Monte Carlo data at low densities and takes a maximum value ${{\boldsymbol{T}}}_{{\bf{c}}}^{{\bf{n}}{\bf{n}}}$= 1.68 MeV at ${\boldsymbol{\rho }}{\boldsymbol{/}}{{\boldsymbol{\rho }}}_{{\bf{0}}}{\boldsymbol{=}}{\bf{0.14}}$ with *ρ*_0_ = 0.17 fm^−3^. Also, the critical temperature ${{\boldsymbol{T}}}_{{\bf{c}}}^{{\bf{n}}{\bf{n}}}$ of the proton superconductivity for *Y*_p_ ≤ 0.2 is substantially suppressed at low densities due to np-pairing fluctuations, and starts to dominate over ${{\boldsymbol{T}}}_{{\bf{c}}}^{{\bf{n}}{\bf{n}}}$ only above ${\boldsymbol{\rho }}{\boldsymbol{/}}{{\boldsymbol{\rho }}}_{{\bf{0}}}{\boldsymbol{=}}{\bf{0.70}}$(0.77) for *Y*_p_ = 0.1(0.2), and (iii) the deuteron condensation temperature ${{\boldsymbol{T}}}_{{\bf{c}}}^{{\bf{d}}}$ is suppressed at *Y*_p_ ≤ 0.2 due to a large mismatch of the two Fermi surfaces.

## Introduction

Superfluidity in strongly interacting Fermi systems has attracted much attention both theoretically and experimentally. For reviews, we refer to refs. ^1,2^ in nuclear physics, refs. ^3–5^ in astrophysics, as well as refs. ^6–10^ in condensed matter physics. It has also been recognized that the dilute neutron matter and two-component ultracold atomic fermions near the unitarity have close similarity. This is due to the strong pairing interactions associated with the large negative neutron-neutron scattering length *a*_s_ = −18.5 fm and relatively small effective range *r*_eff_ = 2.8 fm in the former (see refs. ^6–10^). In the latter atomic system, the pairing interaction can be described by a zero-range potential with a large scattering length^11^. In strongly interacting systems, such as neutron matter and the unitary Fermi gas, effects of pairing fluctuations near the superfluid phase transition are particularly important. Such effects have extensively been studied in cold Fermi gas physics through the observations of various quantities, such as the single-particle excitation spectrum, specific heat, superfluid phase transition temperature (*T*_c_), shear viscosity, and spin susceptibility^10,12,13^. Three of the present authors have recently shown^14^ that a strong coupling theory, being based on the one developed by Nozières and Schmitt-Rink (NSR)^15^ can provide a unified description of neutron matter and an ultracold Fermi gas in the unitary regime. This indicates that the latter atomic gas system can be used as a quantum simulator for neutron star interiors at subnuclear densities.

There are, however, some issues to be overcome for better understanding of the physics of neutron star interiors: Besides neutrons, one should also include a non-zero fraction *Y*_p_ of protons. To deal with this, one needs to extend a strong-coupling theory developed for a two-component atomic Fermi gas to the four-component case involving spin and isospin degrees of freedom. In such a system, not only a neutron-neutron (nn) interaction but also a proton-proton (pp) interaction, as well as a neutron-proton (np) interaction, must be taken into account. In particular, the np interaction in the deuteron channel is stronger than the other interactions, so that it is expected to affect the onset of proton superconductivity. Furthermore, the short-range repulsion of the nuclear force is important to describe the pairing phenomena around the nuclear saturation density. In this paper we will consider all these points, and study the critical temperature of the superfluid phase transitions in asymmetric nuclear matter around the nuclear saturation density *ρ*_0_ = 0.17 fm^−3^, by including the nn, pp and np pairng fluctuations. In this paper, we set $\hslash ={k}_{{\rm{B}}}\mathrm{=1}$, and the system volume is taken to be unity, for simplicity.

## Methods

### Effective hamiltonian

We introduce the pair operator *S*_m_ (${T}_{\ell }$) in the spin-singlet–isospin-triplet (spin-triplet–isospin-singlet) channel with the relative momentum ***k*** and the center of mass momentum ***q***:

1 $${S}_{m}({\boldsymbol{k}},{\boldsymbol{q}})=\sum _{\lambda ,\lambda ^{\prime} }\,\sum _{i,j}\,\langle \frac{1}{2}\frac{1}{2}\lambda \lambda ^{\prime} |00\rangle \langle \frac{1}{2}\frac{1}{2}ij|1m\rangle {c}_{-{\boldsymbol{k}}+{\boldsymbol{q}}\mathrm{/2},\lambda ,i}{c}_{{\boldsymbol{k}}+{\boldsymbol{q}}\mathrm{/2,}\lambda ^{\prime} ,j}$$

2 $${T}_{\ell }({\boldsymbol{k}},{\boldsymbol{q}})=\sum _{\lambda ,\lambda ^{\prime} }\,\sum _{i,j}\langle \frac{1}{2}\frac{1}{2}\lambda \lambda ^{\prime} |1\ell \rangle \langle \frac{1}{2}\frac{1}{2}ij|00\rangle {c}_{-{\boldsymbol{k}}+{\boldsymbol{q}}\mathrm{/2},\lambda ,i}{c}_{{\boldsymbol{k}}+{\boldsymbol{q}}\mathrm{/2},\lambda ^{\prime} ,j}$$

here *c*_*k,λ,i*_ is the fermion annihilation operator with momentum ***k***, spin index *λ*(*λ*′) = ↑, ↓ and isospin index *i* = p, n. The Clebsch-Gordan coefficients in the spin and isospin spaces lead to the projection of the pair operator to appropriate channels.

The effective Hamiltonian in these pairing channels can be written as

3 $$\begin{array}{rcl}H & = & \sum _{{\boldsymbol{p}}}\,\sum _{\lambda =\uparrow ,\downarrow }\,\sum _{i={\rm{p}},{\rm{n}}}\,{\xi }_{{\boldsymbol{p}},i}{c}_{{\boldsymbol{p}},\lambda ,i}^{\dagger }{c}_{{\boldsymbol{p}},\lambda ,i}\\  &  & +\,\frac{1}{2}\sum _{{\boldsymbol{k}},{\boldsymbol{k}}\text{'},{\boldsymbol{q}}}\,[\mathop{\sum }\limits_{m=-\,1}^{+1}\,{S}_{m}^{\dagger }({\boldsymbol{k}},{\boldsymbol{q}}){V}_{{\rm{s}}}({\boldsymbol{k}},{\boldsymbol{k}}{\boldsymbol{^{\prime} }}){S}_{m}({\boldsymbol{k}}{\boldsymbol{^{\prime} }},{\boldsymbol{q}})+\mathop{\sum }\limits_{\ell =-\,1}^{+1}\,{T}_{\ell }^{\dagger }({\boldsymbol{k}},{\boldsymbol{q}}){V}_{{\rm{t}}}({\boldsymbol{k}},{\boldsymbol{k}}{\boldsymbol{^{\prime} }}){T}_{\ell }({\boldsymbol{k}}{\boldsymbol{^{\prime} }},{\boldsymbol{q}})],\end{array}$$

where *V*_s(t)_ is a spin-singlet (triplet) interaction depending on the momentums, ***k*** and ***k***′. ${\xi }_{{\bf{p}},i}=\frac{{{\bf{p}}}^{2}}{2{M}_{i}}-{\mu }_{i}$ is the kinetic energy, measured from the nucleon chemical potentials *μ*_*i*_, and *M*_*i*_ is the nucleon mass. The explicit form of Eq. (3) is given by

4 $$\begin{array}{ccl}H & = & \sum _{{\boldsymbol{p}}}\sum _{\sigma =\uparrow ,\downarrow }\hspace{0.5mm}\sum _{i={\rm{n}},{\rm{p}}}{\xi }_{{\boldsymbol{p}},i}{c}_{{\boldsymbol{p}},\sigma ,i}^{\dagger }{c}_{{\boldsymbol{p}},\sigma ,i}\\  &  & +\sum _{{\boldsymbol{k}}{\boldsymbol{,}}{\boldsymbol{k}}{\boldsymbol{^{\prime} }}{\boldsymbol{,}}{\boldsymbol{q}}}\hspace{0.5mm}\sum _{i={\rm{n}},{\rm{p}}}{V}_{{\rm{s}}}\,({\boldsymbol{k}}{\boldsymbol{,}}{\boldsymbol{k}}{\boldsymbol{^{\prime} }}){c}_{{\boldsymbol{k}}+{\boldsymbol{q}}/2,\uparrow ,i}^{\dagger }{c}_{-{\boldsymbol{k}}+{\boldsymbol{q}}/2,\downarrow ,i}^{\dagger }{c}_{-{\boldsymbol{k}}{\boldsymbol{^{\prime} }}+{\boldsymbol{q}}/2,\downarrow ,i}{c}_{{\boldsymbol{k}}{\boldsymbol{^{\prime} }}+{\boldsymbol{q}}/2,\uparrow ,i}\\  &  & +\sum _{{\boldsymbol{k}}{\boldsymbol{,}}{\boldsymbol{k}}{\boldsymbol{^{\prime} }}{\boldsymbol{,}}{\boldsymbol{q}}}\hspace{0.5mm}\sum _{\sigma =\uparrow ,\downarrow }{V}_{{\rm{t}}}({\boldsymbol{k}}{\boldsymbol{,}}{\boldsymbol{k}}{\boldsymbol{^{\prime} }}){c}_{{\boldsymbol{k}}+{\boldsymbol{q}}/2,\sigma ,{\rm{n}}}^{\dagger }{c}_{-{\boldsymbol{k}}+{\boldsymbol{q}}/2,\sigma ,{\rm{p}}}^{\dagger }{c}_{-{\boldsymbol{k}}{\boldsymbol{^{\prime} }}+{\boldsymbol{q}}/2,\sigma ,{\rm{p}}}{c}_{{\boldsymbol{k}}{\boldsymbol{^{\prime} }}+{\boldsymbol{q}}/2,\sigma ,{\rm{n}}}\\  &  & +\frac{1}{2}\sum _{{\boldsymbol{k}}{\boldsymbol{,}}{\boldsymbol{k}}{\boldsymbol{^{\prime} }}{\boldsymbol{,}}{\boldsymbol{q}}}{V}_{{\rm{s}}}\,({\boldsymbol{k}}{\boldsymbol{,}}{\boldsymbol{k}}{\boldsymbol{^{\prime} }})[{c}_{{\boldsymbol{k}}+{\boldsymbol{q}}/2,\uparrow ,{\rm{n}}}^{\dagger }{c}_{-{\boldsymbol{k}}+{\boldsymbol{q}}/2,\downarrow ,{\rm{p}}}^{\dagger }+{c}_{{\boldsymbol{k}}{\boldsymbol{+}}{\boldsymbol{q}}/2,\uparrow ,{\rm{p}}}^{\dagger }{c}_{-{\boldsymbol{k}}+{\boldsymbol{q}}/2,\downarrow ,{\rm{n}}}^{\dagger }]\\  &  & \times [{c}_{-{\boldsymbol{k}}{\boldsymbol{^{\prime} }}+{\boldsymbol{q}}/2,\downarrow ,{\rm{p}}}{c}_{{\boldsymbol{k}}{\boldsymbol{^{\prime} }}+{\boldsymbol{q}}/2,\uparrow ,{\rm{n}}}+{c}_{-{\boldsymbol{k}}{\boldsymbol{^{\prime} }}+{\boldsymbol{q}}/2,\downarrow ,{\rm{n}}}{c}_{{\boldsymbol{k}}{\boldsymbol{^{\prime} }}+{\boldsymbol{q}}/2\uparrow ,{\rm{p}}}]\\  &  & +\frac{1}{2}\sum _{{\boldsymbol{k}}{\boldsymbol{,}}{\boldsymbol{k}}{\boldsymbol{^{\prime} }}{\boldsymbol{,}}{\boldsymbol{q}}}{V}_{{\rm{t}}}({\boldsymbol{k}}{\boldsymbol{,}}{\boldsymbol{k}}{\boldsymbol{^{\prime} }})[{c}_{{\boldsymbol{k}}+{\boldsymbol{q}}/2,\uparrow ,{\rm{n}}}^{\dagger }{c}_{-{\boldsymbol{k}}+{\boldsymbol{q}}/2,\downarrow ,{\rm{p}}}^{\dagger }-{c}_{{\boldsymbol{k}}{\boldsymbol{+}}{\boldsymbol{q}}/2,\downarrow ,{\rm{p}}}^{\dagger }{c}_{-{\boldsymbol{k}}+{\boldsymbol{q}}/2,\downarrow ,{\rm{n}}}^{\dagger }]\\  &  & \times [{c}_{-{\boldsymbol{k}}{\boldsymbol{^{\prime} }}+{\boldsymbol{q}}/2,\downarrow ,{\rm{p}}}{c}_{{\boldsymbol{k}}{\boldsymbol{^{\prime} }}+{\boldsymbol{q}}/2,\uparrow ,{\rm{n}}}-{c}_{-{\boldsymbol{k}}{\boldsymbol{^{\prime} }}+{\boldsymbol{q}}/2,\downarrow ,{\rm{n}}}{c}_{{\boldsymbol{k}}{\boldsymbol{^{\prime} }}+{\boldsymbol{q}}/2,\uparrow ,{\rm{p}}}].\end{array}$$

### Effective *S*-wave interaction

Throughout this paper, we neglect the isospin symmetry breaking in the interaction *V*_s(t)_, and use the averaged nucleon mass, *M*_p_ = *M*_n_ = *M* = 939 MeV. Furthermore, we only retain the *S*-wave part of *V*_s(t)_ at low energies and introduce a multi-rank separable potential^16–22^

5 $${V}_{\alpha }^{{\rm{SEP}}}(k,k^{\prime} )=\mathop{\sum }\limits_{N=1}^{{N}_{{\rm{\max }}}}\,{\eta }_{\alpha ,N}{\gamma }_{\alpha ,N}(k){\gamma }_{\alpha ,N}(k^{\prime} ),$$

where *γ*_α,N_(*k*) > 0 is a form factor with the suffix *α* = s,t representing the spin-singlet (*α* = s) and spin-triplet (*α* = t) channels, respectively. *η*_α,N_ = ±1 determines the sign of the interaction (e.g., *η*_α,N_ = −1 is attractive). We note that the partial wave expansion of the potential reads ${V}_{\alpha }({\boldsymbol{k}},{\boldsymbol{k}}\mathrm{\text{'})}=4\pi {\sum }_{L,M}{V}_{\alpha }^{(L,M)}(k,k^{\prime} ){Y}_{LM}(\hat{{\boldsymbol{k}}}){Y}_{LM}(\hat{{\boldsymbol{k}}}\text{'})$ with *α* = s(t). Equation (5) is a separable approximation of the *S*-wave contribution, ${V}_{\alpha }^{\mathrm{(0,0)}}(k,k^{\prime} )$. Such a separable potential has been successfully applied to various nuclear systems^14,23–33^.

The simplest case is the rank-one separable potential (SEP1), which is given by setting *j*_max_ = 1 and *η*_α,1_ = −1 in Eq. (5). A typical example of SEP1 is the Yamaguchi potential^16^,

6 $${V}_{\alpha }^{{\rm{SEP}}1}(k,k^{\prime} )={\eta }_{\alpha ,1}{\gamma }_{\alpha \mathrm{,1}}(k){\gamma }_{\alpha \mathrm{,1}}(k^{\prime} )=-\frac{{u}_{\alpha ,1}}{{k}^{2}+{\Lambda }_{\alpha \mathrm{,1}}^{2}}\frac{{u}_{\alpha \mathrm{,1}}}{{k^{\prime} }^{2}+{\Lambda }_{\alpha \mathrm{,1}}^{2}}\mathrm{.}$$

The parameters *u*_α,1_ and Λ_α,1_ are determined such that the observed values of the scattering length and the effective range in the ^1^*S*_0_ channel (*a*_s_, r_s_) = (−18.5 fm, 2.80 fm), and those in the ^3^*S*_1_ channel (*a*_t_, r_t_) = (5.42 fm, 1.76 fm) are reproduced:

7 $${u}_{\alpha \mathrm{,1}}={\Lambda }_{\alpha ,1}^{2}\sqrt{\frac{8\pi }{M}\frac{1}{{\Lambda }_{\alpha ,1}-\mathrm{2/}{a}_{\alpha }}},\,{\Lambda }_{\alpha ,1}=\frac{3+\sqrt{9-16{r}_{\alpha }/{a}_{\alpha }}}{2{r}_{\alpha }}.$$

**Figure 1:**
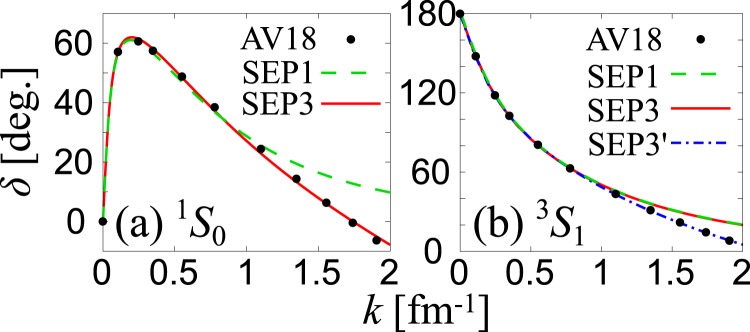
Phase shifts of (**a**) ^1^*S*_0_ neutron-neutron and (**b**) ^3^*S*_1_ neutron-proton interaction. In each figure, black dots show the AV18 phase shift in ref. ^34^. SEP1 and SEP3 represent results of the rank-one and rank-three separable potentials, respectively.

We summarize the evaluated values of *u*_α,1_ and Λ_α,1_ in Table 1, as well as the resulting phase shifts denoted by the dashed lines in Fig. 1(a,b). The filled black circles in the figure represent the phase shifts obtained from the high-precision phenomenological potential, AV18^34^. In the low-momentum region ($k\lesssim 1\,{{\rm{fm}}}^{-1}$), a reasonable agreement between SEP1 and AV18 is obtained in both ^1^*S*_0_ and ^3^*S*_1_ channels, while substantial deviation is seen in the high-momentum region, $k\gtrsim 1$ fm^−1^ in both channels.

**Table 1:** Parameters of rank-one (SEP1) and rank-three (SEP3) separable potentials in ^1^*S*_0_ (*α* = s) and ^3^*S*_1_ (*α* = t) channels.

	*u*_α,1_ [fm^−1^]	*u*_α,2_ [fm^−1^]	*u*_α,3_ [fm^−1^]	Λ_α,1_ [fm^−1^]	Λ_α,2_ [fm^−1^]	Λ_α,3_ [f*m*^−1^]
^1^*S*_0_ (*α* = s, SEP1)	2.6683	0	0	1.1392	—	—
^1^*S*_0_ (*α* = s, SEP3)	4.3097	4.5185	104.82	1.3952	2.3202	3.2578
^3^*S*_1_ (*α* = t, SEP1)	4.4592	0	0	1.4064	—	—
^3^*S*_1_ (*α* = t, SEP3)	4.4619	0.1631	2.2085	1.4064	2.3455	3.0332
^3^*S*_1_ (*α* = t, SEP3′)	6.3578	1.0956	26.814	1.7071	2.9448	2.7045

A better agreement with AV18 in the high momentum region is obtained in the rank-three separable potential (SEP3), which is given by setting *N*_max_ = 3, (*η*_α,1_, *η*_α,2_, *η*_α,3_) = (−1, 1, 1) and the form factors as,

8 $${\gamma }_{\alpha \mathrm{,1}}=\frac{{u}_{\alpha \mathrm{,1}}}{{k}^{2}+{\Lambda }_{\alpha \mathrm{,1}}^{2}},\,{\gamma }_{\alpha \mathrm{,2}}=\frac{{u}_{\alpha \mathrm{,2}}}{{k}^{2}+{\Lambda }_{\alpha ,2}^{2}},\,{\gamma }_{\alpha ,3}=\frac{{u}_{\alpha \mathrm{,3}}{k}^{2}}{{({k}^{2}+{\Lambda }_{\alpha \mathrm{,3}}^{2})}^{2}}.$$

In Table 1, we summarize the SEP3 parameters determined so as to reproduce the AV18 phase shifts in the range 0 fm^−1^ ≤ *k* ≤ 2 fm^−1^, as well as the empirical scattering lengths and effective ranges. As shown in Fig. 1(a), the SEP3 potential (the red line) well reproduces the ^1^*S*_0_ phase shift *δ*, even beyond $k\simeq 1.75$ fm^−1^, where *δ* becomes negative. On the other hand, the SEP3 potential overestimates the phase shift *δ* in the ^3^*S*_1_ channel (the red line) in Fig. 1(b) when $k\gtrsim 1$ fm^−1^.

To further improve the agreement, we introduce a SEP3′ potential for the ^3^*S*_1_ channel with the parameters in Table 1. Here, the AV18 phase shift is fitted in the range 0 fm^−1^ ≤ *k* ≤ 2 fm^−1^, without stringent constraint on the empirical value of *r*_t_. Although the effective range and the deuteron binding energy, in SEP3′ differ from the empirical values by about 9% and 4%, respectively, (see Table 2), one sees in Fig. 1(b) that SEP3′ (blue dash-dotted line) gives good agreement with AV18 to $k\simeq 2$ fm^−1^. In the following, we employ SEP1, SEP3 and SEP3′, to study the superfluid instabilities of asymmetric nuclear matter.

**Table 2:** Scattering lengths *a*_α_, effective ranges *r*_α_, as well as the binding energy *E*_d_ of deuteron for ^3^*S*_1_ channel with the parametrization shown in Table 1.

	*a*_α_ [fm]	*r*_α_ [fm]	*E*_d_ [MeV]
^1^*S*_0_ (*α* = s, SEP1)	−18.50	2.80	—
^1^*S*_0_ (*α* = s, SEP3)	−18.50	2.80	—
^3^*S*_1_ (*α* = t, SEP1)	5.42	1.76	−2.22
^3^*S*_1_ (*α* = t, SEP3)	5.42	1.76	−2.22
^3^*S*_1_ (*α* = t, SEP3′)	5.42	1.91	−2.15

### Thermodynamic potential with pairing fluctuations

**Figure 2:**
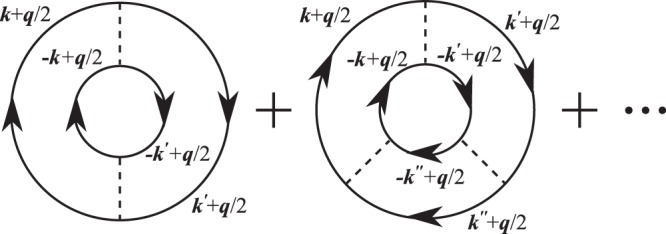
NSR strong-coupling corrections *δ*Ω_NSR_ to the thermodynamic potential Ω in asymmetric nuclear matter at nonzero temperatures. The solid and dashed lines denote the nucleon Green’s function *G*_*i*_ and the bare nucleon-nucleon interaction *V*_α_(***k***, ***k***′), respectively. ***k***, ***k***′, and ***k***″ are relative momenta of nucleons and ***q*** is the center-of-mass momentum of each pair.

We include strong pairing fluctuations originating from *V*_α=s,t_ at finite temperatures within the framework of NSR^15^. In this scheme, the so-called strong-coupling corrections *δ*Ω_NSR_ to the thermodynamic potential Ω are diagrammatically given in Fig. 2. We note that effects of pairing fluctuations for pure neutron matter at zero temperature was previously discussed in ref. ^14^ by using a rank-one separable interaction. Considering the spin-unpolarized nuclear matter, we introduce the one-particle thermal Green’s function in the Hartree approximation, given by

9 $${G}_{{\boldsymbol{p}},i}(i{\omega }_{l})=\frac{1}{i{\omega }_{l}-{\xi }_{{\boldsymbol{p}},i}-{\Sigma }_{i}^{{\rm{H}}}\,({\boldsymbol{p}})}.$$

Here, the Hartree self-energy ${\Sigma }_{i}^{{\rm{H}}}({\boldsymbol{p}})$ involves the contribution from the diagonal force ${V}_{{\rm{D}}}^{{\rm{SEP}}}(k,k)$ in the isospin space originating from the nn and pp interactions, as well as that from the off-diagonal force ${V}_{{\rm{OD}}}^{{\rm{SEP}}}(k,k)$ originating from the np interactions:

10 $${\Sigma }_{i}^{{\rm{H}}}({\boldsymbol{p}})=T\sum _{{\boldsymbol{p}}{\boldsymbol{^{\prime} }},{\omega }_{l}}\,[{V}_{{\rm{D}}}^{{\rm{SEP}}}(k,k){G}_{{\boldsymbol{p}}{\boldsymbol{^{\prime} }},i}(i{\omega }_{l})+{V}_{{\rm{OD}}}^{{\rm{SEP}}}(k,k){G}_{{\boldsymbol{p}}{\boldsymbol{^{\prime} }},\bar{i}}(i{\omega }_{l})],$$

11 $${V}_{{\rm{D}}}^{{\rm{SEP}}}(k,k)={V}_{{\rm{s}}}^{{\rm{SEP}}}(k,k),$$

12 $${V}_{{\rm{OD}}}^{{\rm{SEP}}}(k,k)={V}_{{\rm{t}}}^{{\rm{SEP}}}(k,k)+\frac{1}{2}[{V}_{{\rm{s}}}^{{\rm{SEP}}}(k,k)+{V}_{{\rm{t}}}^{{\rm{SEP}}}(k,k)],$$

where $\bar{i}$ = p(n) for *i* = n(p), *k* = |***p*** − ***p*′|**/2, and *ω*_*l*_ = (2*l* + 1)*πT* is the fermion Matsubara frequency.

Introducing the Fermi momentum distribution for given momentum ***p*** in the Hartree approximation,

13 $${\rho }_{{\boldsymbol{p}},i}^{{\rm{H}}}=T\sum _{{\omega }_{l}}\,{G}_{{\boldsymbol{p}},i}(i{\omega }_{l}),$$

one can write the thermodynamic potential Ω in the NSR theory as,

14 $$\begin{array}{rcl}\Omega  & = & {\Omega }_{{\rm{H}}}+\delta {\Omega }_{{\rm{NSR}}},\\ {\Omega }_{{\rm{H}}} & = & 2T\sum _{{\boldsymbol{p}},i}\,\mathrm{ln}[1+{e}^{-{\xi }_{{\boldsymbol{p}},i}^{{\rm{H}}}/T}]-\sum _{{\boldsymbol{p}},{\boldsymbol{p}}{\boldsymbol{^{\prime} }},i}[{V}_{{\rm{D}}}^{{\rm{SEP}}}(k,k){\rho }_{{\boldsymbol{p}},i}^{{\rm{H}}}{\rho }_{{\boldsymbol{p}}{\boldsymbol{^{\prime} }},i}^{{\rm{H}}}+{V}_{{\rm{OD}}}^{{\rm{SEP}}}(k,k){\rho }_{{\boldsymbol{p}},i}^{{\rm{H}}}{\rho }_{{\boldsymbol{p}}{\boldsymbol{^{\prime} }},\bar{i}}^{{\rm{H}}}],\\ \delta {\Omega }_{{\rm{NSR}}} & = & T\sum _{{\boldsymbol{q}},{\nu }_{l}}\sum _{\alpha }\sum _{m\mathrm{=0,}\pm 1}\,{\rm{Tr}}\,[\mathrm{ln}\,[1+{\hat{\eta }}_{\alpha }{\hat{\Pi }}_{\alpha }^{(m)}\,({\boldsymbol{q}},i{\nu }_{l})]-{\hat{\eta }}_{\alpha }{\hat{\Pi }}_{\alpha }^{(m)}\,({\boldsymbol{q}},i{\nu }_{l}\mathrm{)].}\end{array}$$

Here, $${\xi }_{{\bf{p}},i}^{{\rm{H}}}=\frac{{{\rm{p}}}^{2}}{2M}-{\mu }_{i}+{\Sigma }_{i}^{{\rm{H}}}({\boldsymbol{p}})$$ is the kinetic energy involving the Hartree self-energy $${\Sigma }_{i}^{{\rm{H}}}({\boldsymbol{p}})$$, measured from the chemical potential *μ*_*i*_, and *ν*_*l*_ = 2*πlT* is the boson Matsubara frequency. *δ*Ω_NSR_ in Eq. (14) is the strong-coupling correction to Ω associated with pairing fluctuations in the ^1^*S*_0_ and ^3^*S*_1_ channels, and $${\hat{\eta }}_{\alpha }={\rm{diag}}({\eta }_{\alpha ,1},{\eta }_{\alpha ,2},\ldots ,\,{\eta }_{\alpha ,{N}_{{\rm{\max }}}})$$ Note that Tr is to take over the rank indices, *N*. The *N*_max_ × *N*_max_ matrix pair-correlation function $${\hat{\Pi }}_{\alpha }^{(m)}({\boldsymbol{q}},i{\nu }_{l})=$$$$\{[{\Pi }_{\alpha }^{(m)}({\boldsymbol{q}},i{\nu }_{l}{)]}_{N,{N}^{{\rm{^{\prime} }}}}\}$$ consists of

15 $${[{\hat{\Pi }}_{{\rm{s}}}^{(+\mathrm{1)}}({\boldsymbol{q}},i{\nu }_{l})]}_{N,N^{\prime} }=T\sum _{{\boldsymbol{k}},{\omega }_{l^{\prime} }}\,{\gamma }_{{\rm{s}},N}(k){\gamma }_{{\rm{s}},N^{\prime} }(k){G}_{{\boldsymbol{k}}+{\boldsymbol{q}}\mathrm{/2,}{\rm{p}}}(i{\omega }_{l^{\prime} }+i{\nu }_{l}){G}_{-{\boldsymbol{k}}+{\boldsymbol{q}}\mathrm{/2,}{\rm{p}}}(\,\,-\,i{\omega }_{l^{\prime} }),$$

16 $${[{\hat{\Pi }}_{{\rm{s}}}^{\mathrm{(0)}}({\boldsymbol{q}},i{\nu }_{l})]}_{N,N^{\prime} }=T\sum _{{\boldsymbol{k}},{\omega }_{l^{\prime} }}\,{\gamma }_{{\rm{s}},N}(k){\gamma }_{{\rm{s}},N^{\prime} }(k){G}_{{\boldsymbol{k}}+{\boldsymbol{q}}\mathrm{/2,}{\rm{n}}}(i{\omega }_{l^{\prime} }+i{\nu }_{l}){G}_{-{\boldsymbol{k}}+{\boldsymbol{q}}\mathrm{/2,}{\rm{p}}}(\,\,-\,i{\omega }_{l^{\prime} }),$$

17 $${[{\hat{\Pi }}_{{\rm{s}}}^{(-\mathrm{1)}}({\boldsymbol{q}},i{\nu }_{l})]}_{N,N^{\prime} }=T\sum _{{\boldsymbol{k}},{\omega }_{l^{\prime} }}\,{\gamma }_{{\rm{s}},N}(k){\gamma }_{{\rm{s}},N^{\prime} }(k){G}_{{\boldsymbol{k}}+{\boldsymbol{q}}\mathrm{/2,}{\rm{n}}}(i{\omega }_{l^{\prime} }+i{\nu }_{l}){G}_{-{\boldsymbol{k}}+{\boldsymbol{q}}\mathrm{/2,}{\rm{n}}}(\,\,-\,i{\omega }_{l^{\prime} }),$$

18 $${[{\hat{\Pi }}_{{\rm{t}}}^{\mathrm{(0,}\pm \mathrm{1)}}({\boldsymbol{q}},i{\nu }_{l})]}_{N,N^{\prime} }=T\sum _{{\boldsymbol{k}},{\omega }_{l^{\prime} }}\,{\gamma }_{{\rm{t}},N}(k){\gamma }_{{\rm{t}},N^{\prime} }(k){G}_{{\boldsymbol{k}}+{\boldsymbol{q}}\mathrm{/2,}{\rm{n}}}(i{\omega }_{l^{\prime} }+i{\nu }_{l}){G}_{-{\boldsymbol{k}}+{\boldsymbol{q}}\mathrm{/2,}{\rm{p}}}(\,\,-\,i{\omega }_{l^{\prime} }),$$

where *N*, *N*′ = 1, 2, ..., *N*_max_.

Since we are considering the spin-unpolarized case, Eqs. (15–18) are spin-independent. We briefly note that the first order correction ${\rm{T}}{\rm{r}}[{\hat{\eta }}_{\alpha }{\hat{\Pi }}_{\alpha }^{(m)}({\boldsymbol{q}},i{\nu }_{l})]$ is already involved in the Hartree self-energy ${\varSigma }_{i}^{{\rm{H}}}({\boldsymbol{p}})$^14^, so that we have removed it in Eq. (14) to avoid double counting.

### Critical temperature

The critical temperatures of the ^1^*S*_0_ neutron superfluidity (${T}_{{\rm{c}}}^{{\rm{nn}}}$), ^1^*S*_0_ proton superconductivity (${T}_{{\rm{c}}}^{{\rm{pp}}}$), and ^3^*S*_1_ deuteron condensation (${T}_{{\rm{c}}}^{{\rm{d}}}$), are obtained as functions of baryon density from the Thouless criterion^35^. Here, we introduce the Thouless determinant ${D}_{\alpha }^{(m)}(T)$ defined by

19 $${D}_{{\rm{s}}}^{(-\,\mathrm{1)}}(T)\equiv {\rm{\det }}\,\mathrm{[1}+{\hat{\eta }}_{{\rm{s}}}{\hat{\Pi }}_{{\rm{s}}}^{(-\,\mathrm{1)}}({\boldsymbol{q}}=0,\,i{\nu }_{l}=\mathrm{0)]}=0\,{\rm{at}}\,T={T}_{{\rm{c}}}^{{\rm{nn}}},$$

20 $${D}_{{\rm{s}}}^{(+\mathrm{1)}}(T)\equiv {\rm{\det }}\,\mathrm{[1}+{\hat{\eta }}_{{\rm{s}}}{\hat{\Pi }}_{{\rm{s}}}^{(+\,\mathrm{1)}}({\boldsymbol{q}}=0,i{\nu }_{l}=\mathrm{0)]}=0\,{\rm{at}}\,T={T}_{{\rm{c}}}^{{\rm{pp}}},$$

21 $${D}_{{\rm{t}}}^{\mathrm{(0,}\pm \mathrm{1)}}(T)\equiv {\rm{\det }}\mathrm{[1}+{\hat{\eta }}_{{\rm{t}}}{\hat{\Pi }}_{{\rm{t}}}^{\mathrm{(0,}\pm \mathrm{1)}}\,({\boldsymbol{q}}=\mathrm{0,}\,i{\nu }_{l}=\mathrm{0)]}=0\,{\rm{at}}\,T={T}_{{\rm{c}}}^{{\rm{d}}}.$$

We briefly note that Eqs. (19–21) originate from a “block diagonalized” matrix pair-correlation function with respect to *m* = 0, ±1 so that the Thouless criterion is decomposed into the three Eqs. (19–21). We solve them, together with the particle number equation for the nucleon density,

22 $${\rho }_{i}=-\,\frac{\partial \Omega }{\partial {\mu }_{i}}.$$

In this paper, we approximate ${\Sigma }_{i}^{{\rm{H}}}({\boldsymbol{p}})$ to the value at the Fermi surface (for the theoretical background, see Supplementary Information). Then, we have

23 $${\Sigma }_{i}^{{\rm{H}}}\,({\boldsymbol{p}})\simeq {\Sigma }_{i}^{{\rm{H}}}\,({\boldsymbol{p}}={{\boldsymbol{k}}}_{{\rm{F}},i})\equiv {\overline{\Sigma }}_{i}^{{\rm{H}}},$$

where *k*_F,*i*_ is the nucleon Fermi momentum. Introducing the effective chemical potential

24 $${\mu }_{i}^{{\rm{H}}}\equiv {\mu }_{i}-{\overline{\Sigma }}_{i}^{{\rm{H}}},$$

one can write the particle number equation in the form,

25 $${\rho }_{i}={\rho }_{i}^{{\rm{H}}}+\sum _{i^{\prime} }\,\delta {\rho }_{i^{\prime} }^{{\rm{NSR}}}{L}_{i^{\prime} i},$$

where the Hartree density ${\rho }_{i}^{{\rm{H}}}$ and the NSR correction $\delta {\rho }_{i}^{{\rm{NSR}}}$ are, respectively, given by

26 $${\rho }_{i}^{{\rm{H}}}=2\sum _{{\boldsymbol{p}}}\,{\rho }_{{\boldsymbol{p}},i}^{{\rm{H}}},\,\delta {\rho }_{i}^{{\rm{NSR}}}=-\,\frac{\partial (\delta {\Omega }_{{\rm{NSR}}})}{\partial {\mu }_{i}^{{\rm{H}}}}.$$

The NSR correction $\delta {\rho }_{i}^{{\rm{NSR}}}$ to the number equation involves the diagonal and off-diagonal component of the matrix,

27 $${L}_{ij}={\delta }_{ij}-\frac{{\rm{\partial }}{\bar{\Sigma }}_{i}^{{\rm{H}}}}{{\rm{\partial }}{\mu }_{j}}.$$

This correction naturally arises from *δ*Ω_NSR_, whereas it was ignored in the previous work^23,31,32,36^. We note that *L*_*ij*_ is related to the compressibility matrix ${K}_{ij}^{{\rm{H}}}$ in the mean-field approximation as

28 $${K}_{ij}^{{\rm{H}}}\equiv \frac{{\rm{\partial }}{\rho }_{i}^{{\rm{H}}}}{{\rm{\partial }}{\mu }_{j}}=-\,T\sum _{{\boldsymbol{p}},{\omega }_{l}}{[{G}_{{\boldsymbol{p}},i}(i{\omega }_{l})]}^{2}\,{L}_{ij},$$

which indicates that *L*_*ij*_ corresponds to the vertex correction to the density correlation function. The explicit form of *L*_*ij*_ is given by

29 $$(\begin{array}{cc}{L}_{{\rm{n}}{\rm{n}}} & {L}_{{\rm{n}}{\rm{p}}}\\ {L}_{{\rm{p}}{\rm{n}}} & {L}_{{\rm{p}}{\rm{p}}}\end{array})=\frac{1}{(1+{\kappa }_{{\rm{n}}})(1+{\kappa }_{{\rm{p}}})-{\chi }_{{\rm{n}}}{\chi }_{{\rm{p}}}}(\begin{array}{cc}1+{\kappa }_{{\rm{p}}} & -{\chi }_{{\rm{p}}}\\ -{\chi }_{{\rm{n}}} & 1+{\kappa }_{{\rm{n}}}\end{array}),$$

where

30 $${\kappa }_{i}=-\,T\sum _{{\boldsymbol{p}},{\omega }_{l}}\,{V}_{{\rm{D}}}^{{\rm{S}}{\rm{E}}{\rm{P}}}(\bar{k},\bar{k}){[{G}_{{\boldsymbol{p}},i}(i{\omega }_{l})]}^{2},$$

31 $${\chi }_{i}=-\,T\sum _{{\boldsymbol{p}},{\omega }_{l}}\,{V}_{{\rm{O}}D}^{{\rm{S}}{\rm{E}}{\rm{P}}}(\bar{k},\bar{k}){[{G}_{{\boldsymbol{p}},i}(i{\omega }_{l})]}^{2},$$

with $$\bar{k}$$ = |***k***_F,*i*_ − ***p***|/2.

We note that the particle number density *ρ*_*i*_ is generally obtained from the fully dressed Green’s function ${\mathop{G}\limits^{ \sim }}_{{\boldsymbol{p}},i}(i{\omega }_{\ell })=[i{\omega }_{\ell }-{\xi }_{{\boldsymbol{p}},i}-{\Sigma }_{{\boldsymbol{p}},i}(i{\omega }_{\ell }{)]}^{-1}$ as ${\rho }_{i}=2T{\sum }_{{\boldsymbol{p}},{\omega }_{\ell }}{\mathop{G}\limits^{ \sim }}_{{\boldsymbol{p}},i}(i{\omega }_{\ell })$ with ${\Sigma }_{{\boldsymbol{p}},i}(i{\omega }_{\ell })$ being the full self-energy. On the other hand, *ρ*_*i*_ in the NSR formalism given by Eq. (25) is equivalent to a truncated form of the Dyson series of ${\mathop{G}\limits^{ \sim }}_{{\boldsymbol{p}},i}$:

32 $${\rho }_{i}=2T\sum _{{\boldsymbol{p}},{\omega }_{\ell }}\,[{G}_{{\boldsymbol{p}},i}(i{\omega }_{\ell })+{G}_{{\boldsymbol{p}},i}(i{\omega }_{\ell }){\Sigma }_{{\boldsymbol{p}},i}^{{\rm{N}}{\rm{S}}{\rm{R}}}\,(i{\omega }_{\ell }){G}_{{\boldsymbol{p}},i}(i{\omega }_{\ell })].$$

Here ${\Sigma }_{{\boldsymbol{p}},i}^{{\rm{N}}{\rm{S}}{\rm{R}}}(i{\omega }_{\ell })$ is the self-energy associated with thermal pairing fluctuations,

33 $${\Sigma }_{{\boldsymbol{p}},i}^{{\rm{NSR}}}(i{\omega }_{\ell })=T\sum _{{\boldsymbol{q}},{\nu }_{l}}\,[{T}_{ii}(\tilde{k},\tilde{k},{\boldsymbol{q}},i{\nu }_{l}){L}_{ii}{G}_{{\boldsymbol{q}}-{\boldsymbol{p}},i}(i{\nu }_{l}-i{\omega }_{\ell })+{T}_{i\bar{i}}(\tilde{k},\tilde{k},{\boldsymbol{q}},i{\nu }_{l}){L}_{i\bar{i}}{G}_{{\boldsymbol{q}}-{\boldsymbol{p}},\bar{i}}(i{\nu }_{l}-i{\omega }_{\ell })],$$

where $\mathop{k}\limits^{ \sim }=\frac{{\boldsymbol{q}}}{2}-{\boldsymbol{p}}$. The many-body *T*-matrices *T*_*ij*_(*k*, *k*′, ***q***, *iν*_*l*_) are given by

34 $${T}_{{\rm{pp}}({\rm{nn}})}(k,k^{\prime} ,{\boldsymbol{q}},i{\nu }_{l})=\sum _{N,N^{\prime} }\,{\gamma }_{{\rm{s}},N}(k){[\{{[1+{\hat{\eta }}_{{\rm{s}}}{\hat{\Pi }}_{{\rm{s}}}^{(\pm \mathrm{1)}}({\boldsymbol{q}},i{\nu }_{l})]}^{-1}-1\}{\hat{\eta }}_{{\rm{s}}}]}_{N,N^{\prime} }{\gamma }_{{\rm{s}},N^{\prime} }(k^{\prime} ),$$

35 $$\begin{array}{ccc}{T}_{{\rm{n}}{\rm{p}}({\rm{p}}{\rm{n}})}(k,k{\rm{^{\prime} }},{\boldsymbol{q}},i{\nu }_{l}) & = & \sum _{N,{N}^{{\rm{^{\prime} }}}}\,{\gamma }_{{\rm{s}},N}(k){[\{{[1+{\hat{\eta }}_{{\rm{s}}}{\hat{\Pi }}_{{\rm{s}}}^{(0)}({\boldsymbol{q}},i{\nu }_{l})]}^{-1}-1\}{\hat{\eta }}_{{\rm{s}}}]}_{N,N{\rm{^{\prime} }}}{\gamma }_{{\rm{s}},{N}^{{\rm{^{\prime} }}}}(k{\rm{^{\prime} }})\\  &  & +\sum _{m=0,\pm 1}\hspace{0.5mm}\sum _{N,{N}^{{\rm{^{\prime} }}}}\,{\gamma }_{{\rm{t}},N}(k){[\{{[1+{\hat{\eta }}_{{\rm{t}}}{\hat{\Pi }}_{{\rm{t}}}^{(m)}({\boldsymbol{q}},i{\nu }_{l})]}^{-1}-1\}{\hat{\eta }}_{{\rm{t}}}]}_{N,{N}^{{\rm{^{\prime} }}}}{\gamma }_{{\rm{t}},N{\rm{^{\prime} }}}(k{\rm{^{\prime} }}).\end{array}$$

The asymmetric nuclear matter can conveniently be characterized by the total baryon density *ρ* and the proton fraction *Y*_p_, respectively given by

36 $$\rho ={\rho }_{{\rm{n}}}+{\rho }_{{\rm{p}}},\,{Y}_{{\rm{p}}}=\frac{{\rho }_{{\rm{p}}}}{{\rho }_{{\rm{n}}}+{\rho }_{{\rm{p}}}}.$$

Below, we treat *ρ* and *Y*_p_ as independent parameters, to study their effects on the critical temperatures, ${T}_{{\rm{c}}}^{{\rm{nn}}}$, ${T}_{{\rm{c}}}^{{\rm{d}}}$, and ${T}_{{\rm{c}}}^{{\rm{pp}}}$. We briefly note that, in real neutron star matter, the charge neutrality as well as the chemical equilibrium conditions among protons, neutrons, electrons and muons provide a constraint between *ρ* and *Y*_p_^37^.

## Results and Discussion

**Figure 3:**
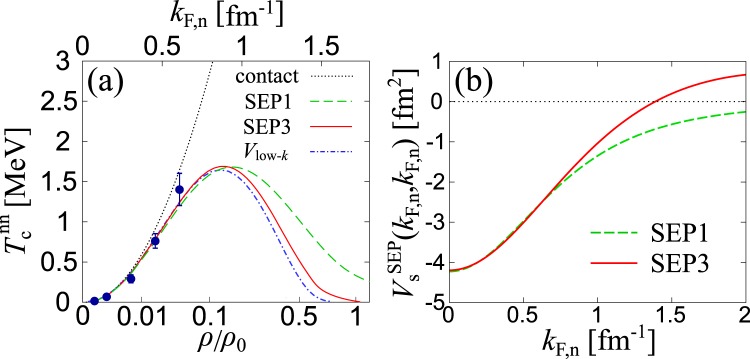
(**a**) Calculated ^1^*S*_0_ neutron superfluid phase transition temperature ${T}_{{\rm{c}}}^{{\rm{nn}}}$ as a function of a nucleon density *ρ* = *ρ*_n_ in pure neutron matter. *k*_F,n_ = (3*π*^2^*ρ*_n_)^1/3^ is the neutron Fermi momentum. The dotted, dashed, and solid lines denote the NSR results of the contact-type (“contact”), rank-one separable (“SEP1”), and rank-three separable (“SEP3”) interactions, respectively. “*V*_low−*k*_” (dot-dashed line) corresponds to the previous NSR work of the renormalization-group based low-momentum interaction^36^. The filled circles represent the result of the lattice Monte-Carlo simulation for the pionless effective field theory^38^. (**b**) The strength of the nn interaction on the Fermi surface, as a function of the neutron Fermi momentum.

We start from the superfluid phase transition temperature ${T}_{{\rm{c}}}^{{\rm{nn}}}$ in pure neutron matter (*Y*_p_ = 0) which has been studied before in different levels of theoretical sophistication. Figure 3(a) shows theoretical estimates of ${T}_{{\rm{c}}}^{{\rm{nn}}}$. We note that since the calculation of ${{T}}_{{\rm{c}}}^{{\rm{n}}{\rm{n}}}$ within SEP3 in the high density region *k*_F,n_ ≳ 1.3 fm^−1^ of pure neutron matter is numerically demanding, we extrapolate them to *k*_F,n_ = 1.73 fm^−1^ where ${{T}}_{{\rm{c}}}^{{\rm{n}}{\rm{n}}}$ invariably disappears because the phase shift at *k* = *k*_F,n_ becomes zero there, by using the Padé approximation. The NSR result of the rank-three separable potential (“SEP3”) shows good agreement with the previous work of NSR with an effective low-momentum interaction *V*_low−*k*_ based on the renormalization group^36^, as well as the result of the lattice Monte-Carlo simulations for the pionless effective field theory^38^ shown by the filled circle (where the interaction is chosen so as to reproduce the nn scattering length and the nn effective range).

To see effects of the effective range and the short-range repulsion in the ^1^*S*_0_ nn channel, we also plot in Fig. 3(a) the calculated ${T}_{{\rm{c}}}^{{\rm{nn}}}$ of NSR with the contact-type interaction *V*_s_(*k*, *k*′) = -*u*_s,1_^2^ (“contact”), where *u*_s,1_ is chosen so as to reproduce *a*_s_, and the rank-one separable interaction (“SEP1”). In the low-density regime (*ρ*/*ρ*_0_ < 0.01) including the neutron drip density ${\rho }_{{\rm{drip}}}/{\rho }_{0}\simeq 1.5\times {10}^{-3}$ (Ref. ^2^), all four theoretical calculations agree well with each other and with the Monte Carlo data, indicating that the critical temperature is determined only by the scattering length. The non-zero effective range (*r*_s_ = 2.8 fm) suppresses ${T}_{{\rm{c}}}^{{\rm{nn}}}$ when $\rho /{\rho }_{0}\gtrsim 0.1$ [see Fig. 3(a)]. It can also be understood as effects of the momentum cut-off Λ_s,1_ associated with the effective range^14,39^. In such a region, the Thouless criterion is approximately given by

37 $$1\simeq {V}_{{\rm{s}}}^{{\rm{S}}{\rm{E}}{\rm{P}}}({k}_{{\rm{F}},{\rm{n}}},{k}_{{\rm{F}},{\rm{n}}})\sum _{{\boldsymbol{k}}}\frac{1}{2{\xi }_{{\boldsymbol{k}},{\rm{n}}}}\,\tanh (\frac{{\xi }_{{\boldsymbol{k}},{\rm{n}}}}{2{T}_{{\rm{c}}}^{{\rm{n}}{\rm{n}}}}).$$

From Eq. (37), one can find that the nn interaction strength on the Fermi surface ${V}_{{\rm{s}}}^{{\rm{SEP}}}({k}_{{\rm{F}},{\rm{n}}},{k}_{{\rm{F}},{\rm{n}}})$ is of importance to evaluate ${T}_{{\rm{c}}}^{{\rm{nn}}}$. Figure 3(b) shows ${V}_{{\rm{s}}}^{{\rm{SEP}}}({k}_{{\rm{F}},{\rm{n}}},{k}_{{\rm{F}},{\rm{n}}})$ of SEP1 and SEP3. Since ${V}_{{\rm{s}}}^{{\rm{SEP}}}(k,k^{\prime} )$ of SEP1 and SEP3 are given by Eqs. (6) and (8), respectively, ${V}_{{\rm{s}}}^{{\rm{SEP}}}({k}_{{\rm{F}},{\rm{n}}},{k}_{{\rm{F}},{\rm{n}}})$ decreases with increasing *k*_F,n_. The decrease of ${V}_{{\rm{s}}}^{{\rm{SEP}}}({k}_{{\rm{F}},{\rm{n}}},{k}_{{\rm{F}},{\rm{n}}})$ is associated with ${\Lambda }_{{\rm{s}}\mathrm{,1}}\simeq \mathrm{3/2}{r}_{{\rm{s}}}$. We briefly note that such a decrease does not occur in the case of the contact-type interaction which is momentum-independent. Moreover, the short-range repulsion of the nn interaction dominates for *ρ*/*ρ*_0_ > 0.54 (near the crust-core transition density $\rho /{\rho }_{0} \sim 0.5$ (Ref. ^3^)) to further suppress ${T}_{{\rm{c}}}^{{\rm{nn}}}$ as *V*_low−*k*_ and SEP3 shown in Fig. 3(a). Indeed, the comparison of SEP1 and SEP3 interactions on the Fermi surface ${V}_{{\rm{s}}}^{{\rm{S}}{\rm{E}}{\rm{P}}}({k}_{{\rm{F}},{\rm{n}}},{k}_{{\rm{F}},{\rm{n}}})$, shown in Fig. 3(b), indicates that the typical strength of the nn interaction decreases with increasing neutron density, and becomes repulsive for *k*_F,n_ > 1.39 fm^−1^. Good agreement of our SEP3 result with the previous *V*_low−*k*_ result over the wide range of baryon density indicates the importance of the detailed interaction structure, as well as associated pairing fluctuations to obtain ${T}_{{\rm{c}}}^{{\rm{nn}}}$.

**Figure 4:**
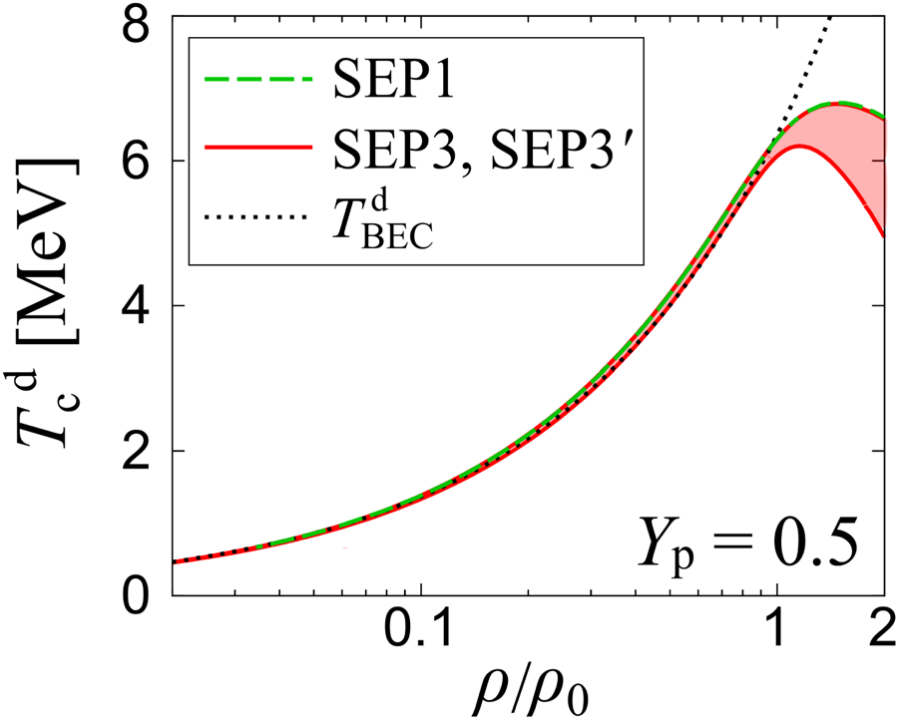
The deuteron condensation temperature ${T}_{{\rm{c}}}^{{\rm{d}}}$ in the ^3^*S*_1_ channel in symmetric nuclear matter (*Y*_p_ = 0.5). The upper and lower bounds of the solid band correspond to the results using the parameter sets shown in Tables 1 and 2, that is, SEP3 and SEP3′, respectively. ${T}_{{\rm{BEC}}}^{{\rm{d}}}$ shows the Bose-Einstein condensation temperature of deuteron gases where the deuteron is approximated as a noninteracting boson.

We proceed to the case of symmetric nuclear matter (*Y*_p_ = 0.5). In this case, examining the Thouless criterion for the nn, pp and np pairing channels, we find that the highest critical temperature is always obtained in the deuteron np channel for *ρ*/*ρ*_0_ ≤ 2. Figure 4 shows the critical temperature of the deuteron condensation, ${T}_{{\rm{c}}}^{{\rm{d}}}$ obtained by SEP3 and SEP3′ for np interaction with SEP3 for nn and pp interactions. The upper (lower) bound of the red solid band corresponds to SEP3 (SEP3′). The green dashed line represents the result of SEP1. For comparison, we also plot in Fig. 4 the Bose-Einstein condensation temperature of an assumed noninteracting deuteron gas, given by^31,32,40^

38 $${T}_{{\rm{B}}{\rm{E}}{\rm{C}}}^{{\rm{d}}}=\frac{\pi }{m}{[\frac{{\rho }_{{\rm{n}}}}{3\zeta (3/2)}\frac{{Y}_{{\rm{p}}}}{1-{Y}_{{\rm{p}}}}]}^{{\textstyle \tfrac{2}{3}}}.$$

The obtained ${T}_{{\rm{c}}}^{{\rm{d}}}$ with all separable interaction potentials approaches ${T}_{{\rm{BEC}}}^{{\rm{d}}}$ in the low-density region. While our result for the symmetric case (*Y*_p_ = 0.5) is qualitatively consistent with the previous work using different separable interactions within the NSR framework^31,32^, ${T}_{{\rm{c}}}^{{\rm{d}}}$ has a peak structure at *ρ*_peak_/*ρ*_0_ > 1, which is in contrast to the previous work giving *ρ*_peak_/*ρ*_0_ = 0.3–0.8 (Refs. ^31,32^). In addition, we do not find a strange back bending behavior of ${T}_{{\rm{c}}}^{{\rm{d}}}$ seen in refs. ^31,32^, irrespective of the use of SEP1, SEP3 and SEP3′. Clarifying these differences remains as our future work. We note that the treatment of the single-particle energy might be a possible origin.

**Figure 5:**
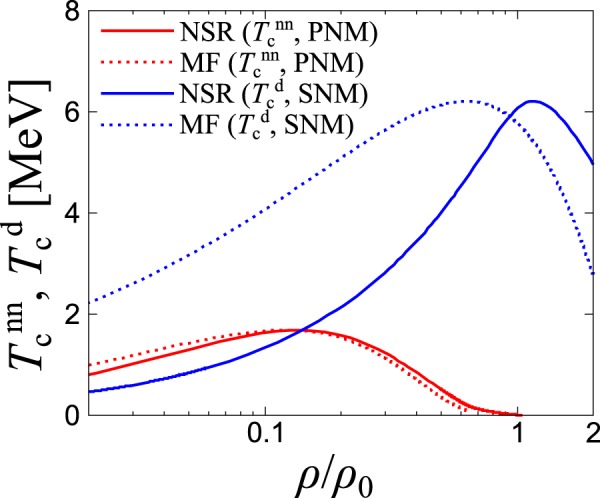
Comparison between the NSR and mean-field (MF) approaches on the critical temperature ${T}_{{\rm{c}}}^{{\rm{nn}}}$ for nn ^1^*S*_0_ pairing in pure neutron matter (PNM) and on ${T}_{{\rm{c}}}^{{\rm{d}}}$ for np ^3^*S*_1_ pairing in symmetric nuclear matter (SNM).

To see the effect of thermal pairing fluctuations on nn and np parings in Figs. 3 and 4, comparisons between the results of NSR and mean-field (MF) approaches for ${T}_{{\rm{c}}}^{{\rm{nn}}}$ in pure neutron matter (*Y*_p_ = 0) and ${T}_{{\rm{c}}}^{{\rm{d}}}$ in symmetric nuclear matter (*Y*_p_ = 0.5) are shown in Fig. 5. For ^1^*S*_0_ nn pairing in pure neutron matter, we observe that the fluctuation effect is not significant, which is consistent with the previous study (Ref. ^36^). In the case of the ^3^*S*_1_ np pairing in symmetric nuclear matter, a substantial modification can be seen, particularly at low densities. This is due to the thermal fluctuation of preformed pairs in the BEC regime which drives ${T}_{{\rm{c}}}^{{\rm{d}}}$ to ${T}_{{\rm{BEC}}}^{{\rm{d}}}$ defined in Eq. (38).

We note here that, both particle number equations and gap equations in our formalism incorporate the effect of quasi-particle correction in terms of the Hartree shift ${\overline{\Sigma }}_{i}^{{\rm{H}}}$. Also the effect of thermal pairing fluctuations is incorporated through the NSR self-energy ${\Sigma }_{{\boldsymbol{p}},i}^{{\rm{N}}{\rm{S}}{\rm{R}}}(i{\omega }_{\ell })$ defined by Eq. (33) in thermodynamic quantities such as the particle number density. However, other quasi-particle corrections such as the effective mass and the wave-function renormalization are not considered in the gap Eqs. (19–21). Also, the induced two-body interactions through density and spin fluctuations are not considered in the gap equations. Possible importance of these effects on the ^3^*SD*_1_ np pairing has been previously discussed in ref. ^41^ at finite temperature and in ref. ^42^ at zero temperature. In Sec. I of Supplemental Information, we also discuss the effective mass M^*^ originating from the ***p***-dependence of ${\Sigma }_{i}^{{\rm{H}}}({\boldsymbol{p}})$ in the dilute neutron matter.

**Figure 6:**
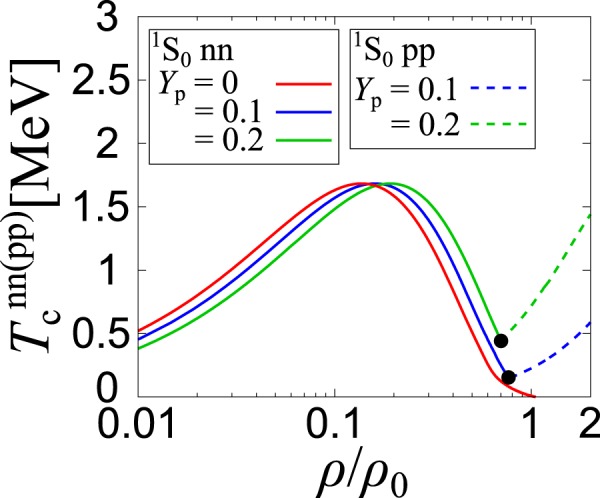
Calculated critical temperatures *T*_c_^nn^ (solid) and ${T}_{{\rm{c}}}^{{\rm{pp}}}$ (dashed) for ^1^*S*_0_ neutron superfluid and proton superconductivity. The circles represent the nucleon densities where both superfluid instabilities simultaneously occur.

We now consider asymmetric nuclear matter within the same theoretical framework. We restrict ourselves to the case with the low proton fraction, ${Y}_{{\rm{p}}}=0.1\, \sim \,0.2$, (which is, however, still valid to the study of the neutron star cooling^37,43,44^). In this range of *Y*_p_, the absolute value of the relative momentum *k* = |***k***| between p and n is smaller than 1.29 fm^−1^, so that we use SEP3 (which gives better agreement with the empirical phase shift at low energies. The Thouless criterion for the nn, pp and np channels gives the highest critical temperature in the nn channel at low densities, while the pp pairing takes over above the nuclear saturation density. Note that, in the low-density limit, ${T}_{{\rm{BEC}}}^{{\rm{d}}}$ becomes dominant even in asymmetric nuclear matter 0 < *Y*_p_ < 0.5 (see Supplementary Information). The deuteron pairing is remarkably suppressed due to imbalanced Fermi surfaces. Figure 6 shows ${T}_{{\rm{c}}}^{{\rm{nn}}}$ and ${T}_{{\rm{c}}}^{{\rm{pp}}}$ in the case of SEP3^37^.

In Fig. 6, with increasing the proton fraction *Y*_p_, the peak of ${T}_{{\rm{c}}}^{{\rm{nn}}}$ is found to gradually move to higher density. This is simply because the neutron density decreases as *ρ*_n_ = (1 − *Y*_p_)*ρ*, so that the whole curve of ${T}_{{\rm{c}}}^{{\rm{nn}}}$ shifts to the right. The black circle in Fig. 6 indicates the density at which ${T}_{{\rm{c}}}^{{\rm{pp}}}$ exceeds ${T}_{{\rm{c}}}^{{\rm{nn}}}$ when *Y*_p_ > 0. Beyond this, the pp interaction becomes more attractive, due to relatively small proton Fermi momentum *k*_F,p_ = (3*π*^2^*ρ*_p_)^1/3^ = (3*π*^2^*ρY*_p_)^1/3^, while the nn interaction is strongly suppressed by the short-range repulsion due to large neutron Fermi momentum *k*_F,n_ = (3*π*^2^*ρ*_n_)^1/3^ = [3*π*^2^*ρ*(1 − *Y*_p_)]^1/3^. At higher density, ${T}_{{\rm{c}}}^{{\rm{pp}}}$ would also be suppressed, but it is beyond the applicability of the present formalism (see Supplementary Information).

**Figure 7:**
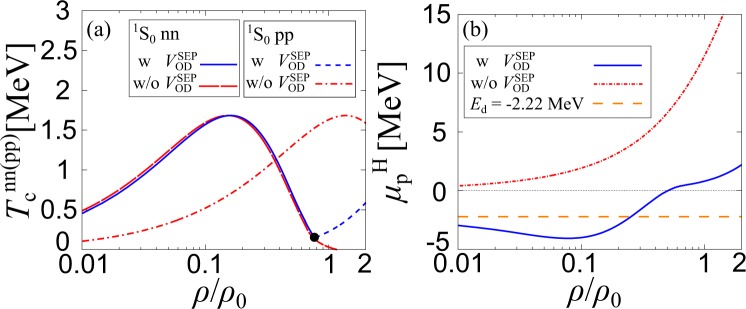
(**a**) The critical temperatures ${T}_{{\rm{c}}}^{{\rm{nn}}}$^(pp)^, and (**b**) the effective proton chemical potential ${\mu }_{{\rm{p}}}^{{\rm{H}}}={\mu }_{{\rm{p}}}-{\overline{\Sigma }}_{{\rm{p}}}^{{\rm{H}}}$, at *Y*_p_ = 0.1 with and without the off-diagonal np interaction ${V}_{{\rm{OD}}}^{{\rm{SEP}}}$. The horizontal dashed line in panel (b) represents the deuteron binding energy *E*_d_ = −2.22 MeV.

To see effects of strong np interactions, we plot the critical temperatures ${T}_{{\rm{c}}}^{{\rm{nn}}}$, as well as, ${T}_{{\rm{c}}}^{{\rm{pp}}}$ in Fig. 7(a). We also show the effective proton chemical potential ${\mu }_{{\rm{p}}}^{{\rm{H}}}$ which is defined in Eq. (24) (at *T* = ${T}_{{\rm{c}}}^{{\rm{nn}}}$, below 0.77*ρ*_0_ and at *T* = ${T}_{{\rm{c}}}^{{\rm{pp}}}$ above 0.77*ρ*_0_), with and without the np interaction, ${V}_{{\rm{OD}}}^{{\rm{SEP}}}$ in Fig. 7(b). We find that while ${T}_{{\rm{c}}}^{{\rm{nn}}}$ is insensitive to the strength of the np interaction, ${T}_{{\rm{c}}}^{{\rm{pp}}}$ is substantially affected. The latter can be understood by the behavior of ${\mu }_{{\rm{p}}}^{{\rm{H}}}$ When ${V}_{{\rm{OD}}}^{{\rm{SEP}}}$ = 0, ${\mu }_{{\rm{p}}}^{{\rm{H}}}$ is always positive as shown in Fig. 7(b), indicating that the proton Fermi surface is formed, irrespective of the value of baryon density *ρ*, naturally leading to the proton superconductivity. On the other hand, when ${V}_{{\rm{OD}}}^{{\rm{SEP}}}$≠0, the strong np interaction in the deuteron channel reduces ${\mu }_{{\rm{p}}}^{{\rm{H}}}$ in the low-density region, to eventually approach the deuteron binding energy *E*_d_ = −2.22 MeV in the low-density limit. As a result, pp pairing does not take place in this regime. In the low density limit with 0 < *Y*_p_ < 0.5, one finds ${\mu }_{{\rm{n}}} \sim {\mu }_{{\rm{n}}}^{{\rm{H}}}\to 0$ and ${\mu }_{{\rm{p}}} \sim {\mu }_{{\rm{p}}}^{{\rm{H}}}\to {E}_{{\rm{d}}}$^40^ as in the case of an asymmetric two-component Fermi atomic gas^45^.

**Figure 8:**
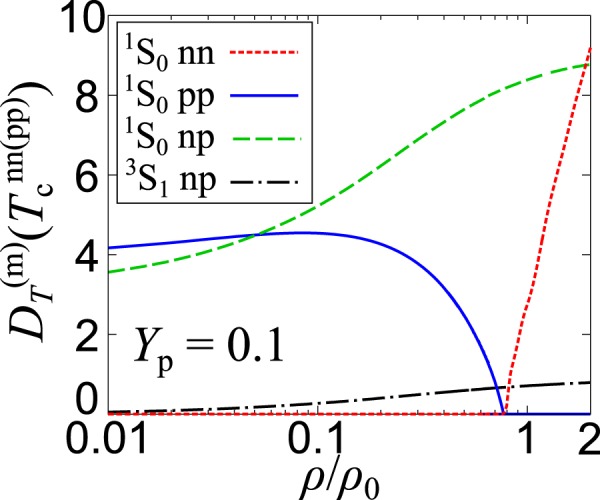
Thouless determinants, ${D}_{\alpha }^{(m)}$ in all four channels as functions of the baryon density *ρ* with *Y*_p_ = 0.1 at *T* = ${T}_{{\rm{c}}}^{{\rm{nn}}}$ below *ρ* = 0.77*ρ*_0_ and at *T* = ${T}_{{\rm{c}}}^{{\rm{pp}}}$ above *ρ* = 0.77*ρ*_0_. The dotted, solid, dashed, and dot-dashed lines represent ${D}_{\alpha }^{(m)}$ of the ^1^*S*_0_ nn, ^1^*S*_0_ pp, ^1^*S*_0_ np, and ^3^*S*_1_ np channels, respectively.

Figure 8 shows the Thouless determinants ${D}_{\alpha }^{(m)}(T)$ in Eqs. (19–21) for *Y*_p_ = 0.1 at *T* = ${T}_{{\rm{c}}}^{{\rm{nn}}}$ below 0.77*ρ*_0_, and at *T* = ${T}_{{\rm{c}}}^{{\rm{pp}}}$ above 0.77*ρ*_0_. When ${D}_{\alpha }^{(m)}(T)$ becomes smaller to vanish, pairing fluctuations become stronger and eventually diverge at the second-order superfluid/superconducting phase transition. Such diverging fluctuations can be seen in the ^1^*S*_0_ nn channel for *ρ* < 0.77*ρ*_0_, as well as in the ^1^*S*_0_ pp channel for *ρ* > 0.77*ρ*_0_. On the other hand, pairing fluctuations in the ^1^*S*_0_ np channel are weak, compared to the other channels. The Thouless determinant in the ^3^*S*_1_ np channel is close to zero over the entire density, but the deuteron condensation does not occur when *Y*_p_ = 0.1, because of the large difference of the chemical potentials between neutrons and protons. Nevertheless, strong pairing fluctuations in the deuteron channel play a crucial role for ${T}_{{\rm{c}}}^{{\rm{pp}}}$, as seen in Fig. 7.

**Figure 9:**
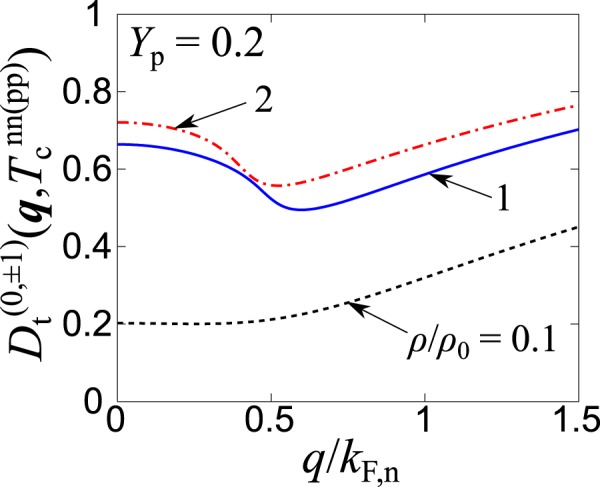
Thouless determinant in the deuteron channel as a function of the center-of-mass momentum *q* at *Y*_p_ = 0.2 for different values of the baryon density.

Before ending this section, we discuss the possibility of a Fulde-Ferrell-Larkin-Ovchinnikov (FFLO) state^46–49^ in the deuteron channel for 0 < *Y*_p_ < 0.2 (which is relevant for neutron stars). The FFLO state may occur, when two kinds of fermions attractively interact with each other in the presence of population imbalance. In such a case, the Cooper pairs with a non-zero center-of-mass momentum are formed. In the present case, the Thouless determinant at a non-zero momentum^50,51^, ${D}_{{\rm{t}}}^{(0,\pm 1)}({\boldsymbol{q}},T)={\rm{\det }}[1+{\hat{\eta }}_{{\rm{t}}}{\hat{\Pi }}_{{\rm{t}}}^{(0,\pm 1)}({\boldsymbol{q}},i{\nu }_{l}=0)]$ is an appropriate measure. Figure 9 shows the center-of-mass momentum (*q* = |***q***|) dependence of ${D}_{{\rm{t}}}^{(0,\pm 1)}({\boldsymbol{q}},T)$ at $T={T}_{{\rm{c}}}^{{\rm{nn}}({\rm{pp}})}$ in asymmetric nuclear matter with *Y*_p_ = 0.2. We find that ${D}_{{\rm{t}}}^{(0,\pm 1)}({\boldsymbol{q}},T)$ takes a minimum at a non-zero momentum q^*^ in the high-density region (*ρ* > *ρ*_0_). Indeed, q^*^ at *ρ* = *ρ*_0_ in Fig. 9 is close to the typical momentum of the FFLO pairing, ${k}_{{\rm{F}},{\rm{n}}}^{{\rm{e}}{\rm{f}}{\rm{f}}}-{k}_{{\rm{F}},{\rm{p}}}^{{\rm{e}}{\rm{f}}{\rm{f}}}={(2m{\mu }_{{\rm{n}}}^{{\rm{H}}})}^{1/2}-{(2{\rm{m}}{\mu }_{{\rm{p}}}^{{\rm{H}}})}^{1/2}\simeq 0.7{k}_{{\rm{F}},{\rm{n}}}$. Although ${D}_{{\rm{t}}}^{(0,\pm 1)}({{\boldsymbol{q}}}^{\ast },T)$ is still far away from zero, it may be interpreted as a precursor of the FFLO state at larger *Y*_p_.

## Conclusion

In this paper, we have extended the Nozières-Schmitt-Rink approach to four-component fermion system, to examine the superfluid phase transition at finite temperatures in asymmetric nuclear matter at nuclear and subnuclear densities. Including pairing fluctuations in the *S*-wave neutron-neutron, proton-proton, and neutron-proton channels, we evaluated the critical temperature of ^1^*S*_0_ neutron superfluidity ${T}_{{\rm{c}}}^{{\rm{nn}}}$ and proton superconductivity ${T}_{{\rm{c}}}^{{\rm{pp}}}$. We clarified effects of strong neutron-proton pairing fluctuations in the deuteron channel. While resultant ${T}_{{\rm{c}}}^{{\rm{nn}}}$ in pure neutron matter agrees well with the previous Monte Carlo data in the low baryon-density region, it is remarkably suppressed around the nuclear saturation density *ρ*_0_, due to the short-range nn repulsion. We found that ${T}_{{\rm{c}}}^{{\rm{pp}}}$ at low-density is substantially suppressed by the neutron-proton pairing fluctuations.

There are several future directions to be explored on the basis of the framework developed in this paper.

1. We have focused on the superfluid/superconducting instability in the normal phase throughout the paper. However, the present model together with the framework of ref. ^14^ can be combined to study the superfluid phase below the critical temperature, such as equation of state, as well as magnitude of the pairing gap.
2. To improve the accuracy of ${T}_{{\rm{c}}}^{{\rm{nn}},{\rm{pp}},{\rm{d}}}$, we need to include the coupled ^3^*S*_1_-^3^*D*_1_ channel potential beyond the present ^3^*S*_1_ channel potential. Such a channel-coupling introduces extra in-medium effect associated with the Pauli blocking by the intermediate ^3^*D*_1_ state.
3. There are correlations which are ignored in the present paper, such as Gorkov and Melik-Barkhudarov (GMB) screening^52–55^, as well as the competition between the screening and anti-screening corrections^42,56,57^.
4. The nn pairing in the ^3^*P*_2_ channel^3,58–60^ would cause a dominant superfluid component in the liquid core of neutron stars. Introducing a separable interaction in the *P*-wave channel and applying the present framework would be a first step toward the analysis of such unconventional superfluids.
5. Although we have used separable form of the nucleon-nucleon potential to study the effects of paring fluctuations, one could also apply more systematic chiral effective nucleon-nucleon interaction^61^ to our approach.

## Supplementary information

Supplementary materials
