# Supplementary material for: Superfluid Phase Transitions and Effects of Thermal Pairing Fluctuations in Asymmetric Nuclear Matter
Source: Sci Rep. 2019 Dec 6;9:18477. doi: 10.1038/s41598-019-54010-7 (PMC6897924; doi:10.1038/s41598-019-54010-7)
Supplement: Supplementary file 1 — Supplementary materials [file 41598_2019_54010_MOESM1_ESM.pdf]

Supplementary Information for "Superfluid Phase Transition and Effects of Thermal Pairing Fluctuations in Asymmetric Nuclear Matter"

Hiroyuki Tajima, Tetsuo Hatsuda, Pieter van Wyk, and Yoji Ohashi

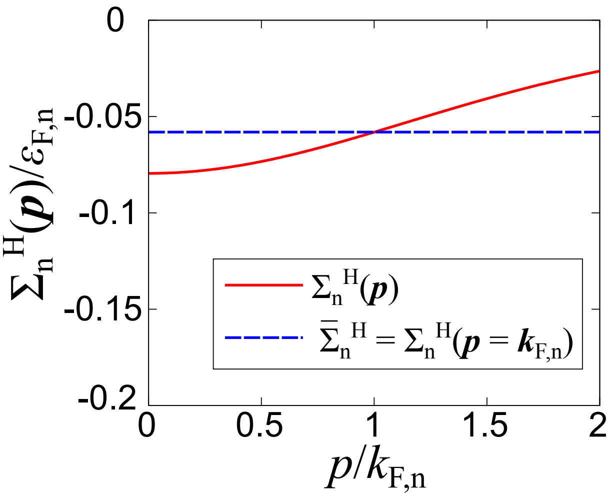

FIG. 1: Hartree self-energy $\Sigma_{\rm n}^{\rm H}(\boldsymbol{p})$ and the approximated Hartree shift $\bar{\Sigma}_{\rm n}^{\rm H} = \Sigma_{\rm n}^{\rm H}(\boldsymbol{p} = \boldsymbol{k}_{\rm F,n})$ in pure neutron matter with SEP at $\rho = 0.29\rho_0$, $\varepsilon_{\rm F,n} = \mu_{\rm n}$ and $T = 0.1\varepsilon_{\rm F,n}$

I. THE HARTREE SHIFT

Figure 1 shows the momentum dependence of the Hartree self-energy $\Sigma_i^{\rm H}(\boldsymbol{p})$ in the pure neutron matter at $\rho = 0.29\rho_0$ with SEP1. We set $\varepsilon_{\rm F,n} = \mu_{\rm n}$ and $T = 0.1\varepsilon_{\rm F,n}$, and pairing-fluctuation effects are neglected for simplicity. The magnitude of the Hartree shift is relatively small compared to the neutron chemical potential $\mu_{\rm n}$ and its momentum dependence is not substantial. Since the momentum at the Fermi surface is the most important for Cooper pairings, we introduce an approximation $\bar{\Sigma}_i^{\rm H} = \Sigma_i^{\rm H}(\boldsymbol{p} = k_{{\rm F},i})$ as adopted in the text.

In general, the momentum dependence of the Hartree self-energy near the Fermi surface gives rise to the effective mass M^* defined by [1]

$$\frac{1}{M^*} = \frac{1}{M} + 2\left.\frac{\partial \Sigma_i^{\rm H}(\boldsymbol{p})}{\partial p^2}\right|_{\boldsymbol{p}=\boldsymbol{k}_{{\rm F},i}}. \tag{1}$$

From Fig. 1, we find $M^* \simeq 0.98M$. In the present work, we have not taken into account this small correction.

We note that the present approximation of the Hartree shift is different from the previous work [2], where $\bar{\Sigma}_{\rm n}^{\rm H} = V_{\rm s}(\boldsymbol{0},\boldsymbol{0})\rho_{\rm n}^{\rm H}/2 = V_{\rm s}^{\rm SEP}(0,0)\rho_{\rm n}^{\rm H}/2$ is used. While such an approximation of the Hartree shift is sufficient enough in the low-density region, it leads to the divergence of L_{ij} near the nuclear saturation density. Furthermore, the present form of the Hartree shift is rather consistent with the mean-field approximation under the separable interac-

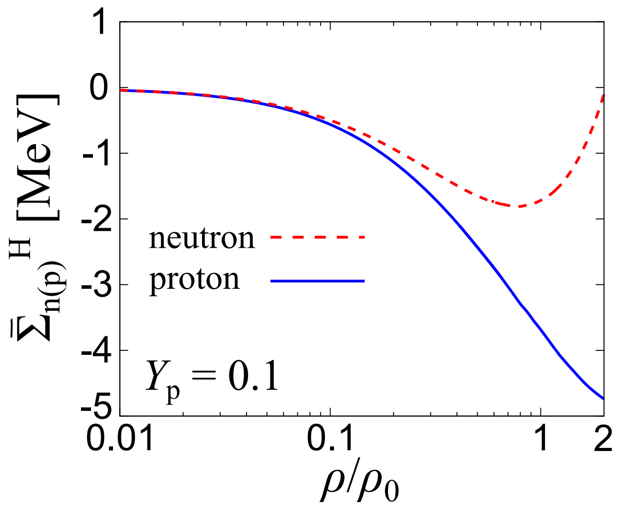

FIG. 2: The approximate self-energy shifts $\bar{\Sigma}_i^{\mathrm{H}}$ at $Y_{\mathrm{p}} = 0.1$.

tion $V_{\mathrm{s}}^{\mathrm{SEP}}(k, k')$.

Figure 2 shows the baryon density dependence of $\bar{\Sigma}_i^{\mathrm{H}}$ in asymmetric nuclear matter with $Y_{\mathrm{p}} = 0.1$. In the low-density limit, the shifts are negligibly small where the interaction can be well approximated by the contact-type interaction. While $\bar{\Sigma}_{\mathrm{n}}^{\mathrm{H}}$ increases around the nuclear matter density due to the short-range repulsion in the $^1S_0$ nn channel, $\bar{\Sigma}_{\mathrm{p}}^{\mathrm{H}}$ decreases further, reflecting the difference between $k_{\mathrm{F,p}}$ and $k_{\mathrm{F,n}}$. In addition, the behavior of $\bar{\Sigma}_{\mathrm{p}}^{\mathrm{H}}$ is mainly dictated by the $^3S_1$ np interaction rather than the $^1S_0$ pp interaction because of $\rho_{\mathrm{n}} > \rho_{\mathrm{p}}$ in neutron star matter.

II. $T_{\mathrm{c}}^{\mathrm{pp}}$ AND $T_{\mathrm{c}}^{\mathrm{d}}$ AT HIGHER AND LOWER DENSITIES

Since our separable interactions are adjusted so as to reproduce the AV18 phase shift up to $k = 2\ \ \mathrm{fm}^{-1}$, they cannot be used to investigate the properties of neutron matter above $\rho = 1.59\rho_0$ (where $k_{\mathrm{F,n}} = 2\ \mathrm{fm}^{-1}$). On the other hand, the effective pp interaction $V_{\mathrm{s}}^{\mathrm{SEP}}(k_{\mathrm{F,p}}, k_{\mathrm{F,p}})$ at the proton Fermi momentum $k_{\mathrm{F,p}}$ is still in the range of $0 \leq k_{\mathrm{F,p}} \leq 2\ \mathrm{fm}^{-1}$ even up to $\rho = 15.9\rho_0$ in the case of $Y_{\mathrm{p}} = 0.1$. Therefore, just to see the qualitative behavior at high density, we plot $T_{\mathrm{c}}^{\mathrm{pp}}$ up to $5\rho_0$ in Fig .3. The result exhibits an upturn behavior in higher density regime due to the effective-range correction as well as short-range repulsion in the $^1S_0$ pp channel. The $^3S_1$ np interaction modifies its density dependence through the suppression of the effective proton chemical potential $\mu_{\mathrm{p}}^{\mathrm{H}}$.

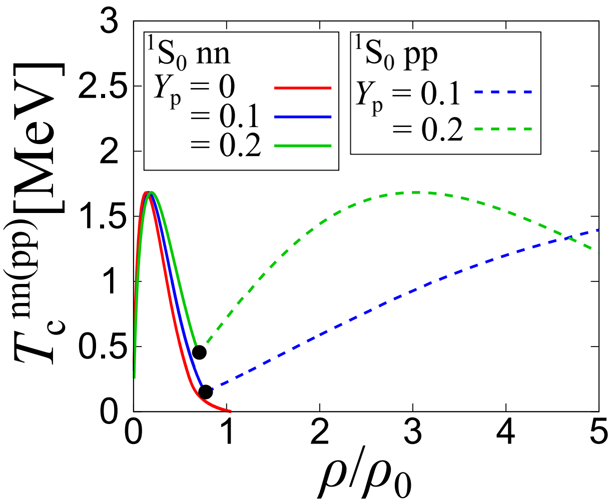

FIG. 3: Calculated critical temperature $T_{\rm c}^{\rm nn(pp)}$ of the $^1S_0$ neutron superfluidity (proton superconductivity) in asymmetric nuclear matter up to $\rho = 5\rho_0$.

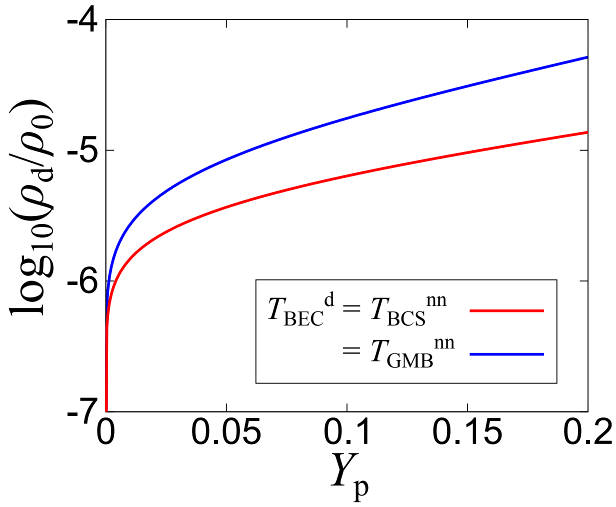

FIG. 4: The proton fraction dependence of the critical nucleon density $\rho_{\rm d}$ where $T_{\rm BEC}^{\rm d} = T_{\rm BCS}^{\rm nn}$ and $T_{\rm BEC}^{\rm d} = T_{\rm GMB}^{\rm nn}$.

On the other hand, in the low-density limit, $T_{\rm BEC}^{\rm d}$ exceeds $T_{\rm c}^{\rm nn}$ in the case of a finite proton fraction. In this limit, $T_{\rm c}^{\rm nn}$ is given by the zero-range BCS result

$$T_{\rm BCS}^{\rm nn} = \frac{8e^{\gamma}}{\pi e^2}\varepsilon_{\rm F,n}e^{\frac{\pi}{2k_{\rm F,n}a_{\rm s}}}. \tag{2}$$

where $\gamma = 0.577$ is the Euler constant. Since $T_{\rm c}^{\rm d}$ is equal to $T_{\rm BEC}^{\rm d}$ due to the large deuteron binding energy $|E_{\rm d}| = 2.22$ MeV, we can analytically obtain the critical nucleon density $\rho_{\rm d}$

where $T^{\rm d}_{\rm BEC} = T^{\rm nn}_{\rm BCS}$ as

$$\rho_{\rm d} = \frac{\pi}{24a_{\rm s}^3(1-Y_{\rm p})}\left[2\ln\left(\frac{\pi}{2}\right) + 2 - \gamma + \frac{2}{3}\ln\left(\frac{1}{9\pi^2\zeta(3/2)}\frac{Y_{\rm p}}{1-Y_{\rm p}}\right)\right]^{-3}. \tag{3}$$

Figure 4 shows the proton fraction dependence of $\rho_{\rm d}$. We also plot $\rho_{\rm d}$ obtained from the GMB result $T^{\rm d}_{\rm BEC} = T^{\rm nn}_{\rm GMB} = (4e)^{-1/3}T^{\rm nn}_{\rm BCS}$ in the presence of the screening correction [3]. In the relevant region for a neutron star ($0 < Y_{\rm p} < 0.2$), $\rho_{\rm d}$ is smaller than the neutron drip density $\rho_{\rm drip}/\rho_0 = 1.5 \times 10^{-3}$ [4]. We note that Eq. (3) is valid at small proton fraction ($Y_{\rm p} < 0.2$), where $\rho_{\rm d}$ appears in the sufficiently low-density regime $[(k_{\rm F,n}a_{\rm s})^{-1} < -1]$ [5].

---

[1] M. Jin, M. Urban, and P. Schuck, Phys. Rev. C **82**, 024911 (2010).

[2] P. van Wyk, H. Tajima, D. Inotani, A. Ohnishi, and Y. Ohashi, Phys. Rev. A **97**, 013601 (2018).

[3] L. P. Gorkov and T. K. Melik-Barkhudarov, Sov. Phys. JETP **13**, 1018 (1961) [Zh. Eksp. Teor. Fiz. **40**, 1452 (1961)].

[4] D.-J. Dean and M. Hjorth-Jensen, Rev. Mod. Phys. **75**, 607 (2003).

[5] H. Tajima, T. Hatsuda and Y. Ohashi, J. Phys.: Conf. Ser. **969**, 012003 (2018).
